# Phospholipase Cδ1 suppresses cell migration and invasion of breast cancer cells by modulating KIF3A-mediated ERK1/2/β- catenin/MMP7 signalling

**DOI:** 10.18632/oncotarget.16072

**Published:** 2017-03-10

**Authors:** Qing Shao, Xinrong Luo, Dejuan Yang, Can Wang, Qiao Cheng, Tingxiu Xiang, Guosheng Ren

**Affiliations:** ^1^ Chongqing Key Laboratory of Molecular Oncology and Epigenetics, The First Affiliated Hospital of Chongqing Medical University, Chongqing, China; ^2^ Department of Endocrine and Breast Surgery, The First Affiliated Hospital of Chongqing Medical University, Chongqing, China

**Keywords:** PLCD1, tumour suppressor, breast cancer, KIF3A, MMP7

## Abstract

Phospholipase C δ1 (*PLCD1*) encodes an enzyme involved in energy metabolism, calcium homeostasis and intracellular movement. It is located at 3p22 in a region that is frequently deleted in multiple cancers, and the PLCD1 enzyme is a potential tumour suppressor in breast cancer that inhibits matrix metalloprotease (MMP) 7, but the detailed mechanism remains elusive. In this study, we found that PLCD1 was downregulated in breast cancers, and the gain-or-loss functional assay revealed that PLCD1 inhibited cell migration and invasion *in vitro* via the ERK1/2/β-catenin/MMP7 signalling pathway. Furthermore, KIF3A was identified as a downstream mediator of PLCD1, and there was an inverse correlation between the expression of PLCD1 and KIF3A. Knockdown of KIF3A expression alone suppressed cell migration and invasion, and attenuated ERK1/2/β-catenin/MMP7 signalling that was reactivated by knocking down PLCD1 *in vitro*. Collectively, our findings suggest that PLCD1 acts as a tumour suppressor, by KIF3A-mediated suppression of ERK1/2/β-catenin/MMP7 signalling, at least in part, in breast cancer.

## INTRODUCTION

Breast cancer is the leading cause of cancer-associated death in women worldwide [1], mainly due to its invasiveness and propensity for metastasis, both of which are important hallmarks of cancer [2]. A number of signalling pathways are involved in this process, including the Wnt/β-catenin signal pathway, and they can be in a constant state of activation following dysregulation of cancer genes and/or the inactivation of tumour suppressor genes (TSGs) [3].

The 3p22 region, which includes or is close to a number of TSGs such as *PLCD1*, *FHIT*, *RASSF1A*, and *DLEC1*, is frequently deleted in multiple cancers [4]. Several candidate TSGs implicated in aerodigestive tumours have been identified through extensive expression profiling and epigenetic characterization, including *BLU*, *DLEC1* and *PLCD1* [5]. PLCD1 has also been identified as a TSG in gastric cancer [5], oesophageal squamous cell carcinoma [6], KRAS-mutated colorectal cancer [7], chronic myeloid leukaemia [8], and breast cancer in our previous work [9, 10].

Phospholipase C (PLC) is a key enzyme in the phosphoinositide metabolism system [11]. This enzyme hydrolyses phosphatidylinositol 4,5-bisphosphate (PI(4,5) P2) to generate two second messengers, inositol-1,4,5-trisphoaphate (IP3) and 1,2-diacylglycerol (DAG), and these increase intracellular Ca^2+^ levels and activate protein kinase (PKC) signalling pathways, respectively. Phosphoinositide metabolism is involved in tissue differentiation, cytoskeleton transformation, tumorigenesis and many other physiological processes. Based on structure and regulatory activation mechanisms, 13 isozymes of PLC have been identified and divided into β, γ, δ, ε, ζ and η subtypes [12]. PLCD1 belongs to the PLCδ subgroup, which is considered the basic isoform of the PLC family [13, 14].

Anti-tumour effects have been reported for PLCD1 in multiple cancers. However, the detailed mechanism of action remains poorly understood. In the present study, expression of PLCD1 in primary breast cancers was investigated. Tumour suppression activity was validated *in vitro*, and the KIF3A-mediated ERK1/2/β-catenin/MMP7 signalling pathway was demonstrated to mediate the anti-tumour function, at least in part.

## RESULTS

### PLCD1 is downregulated in breast cancer cell lines and tumours

**Figure 1:**
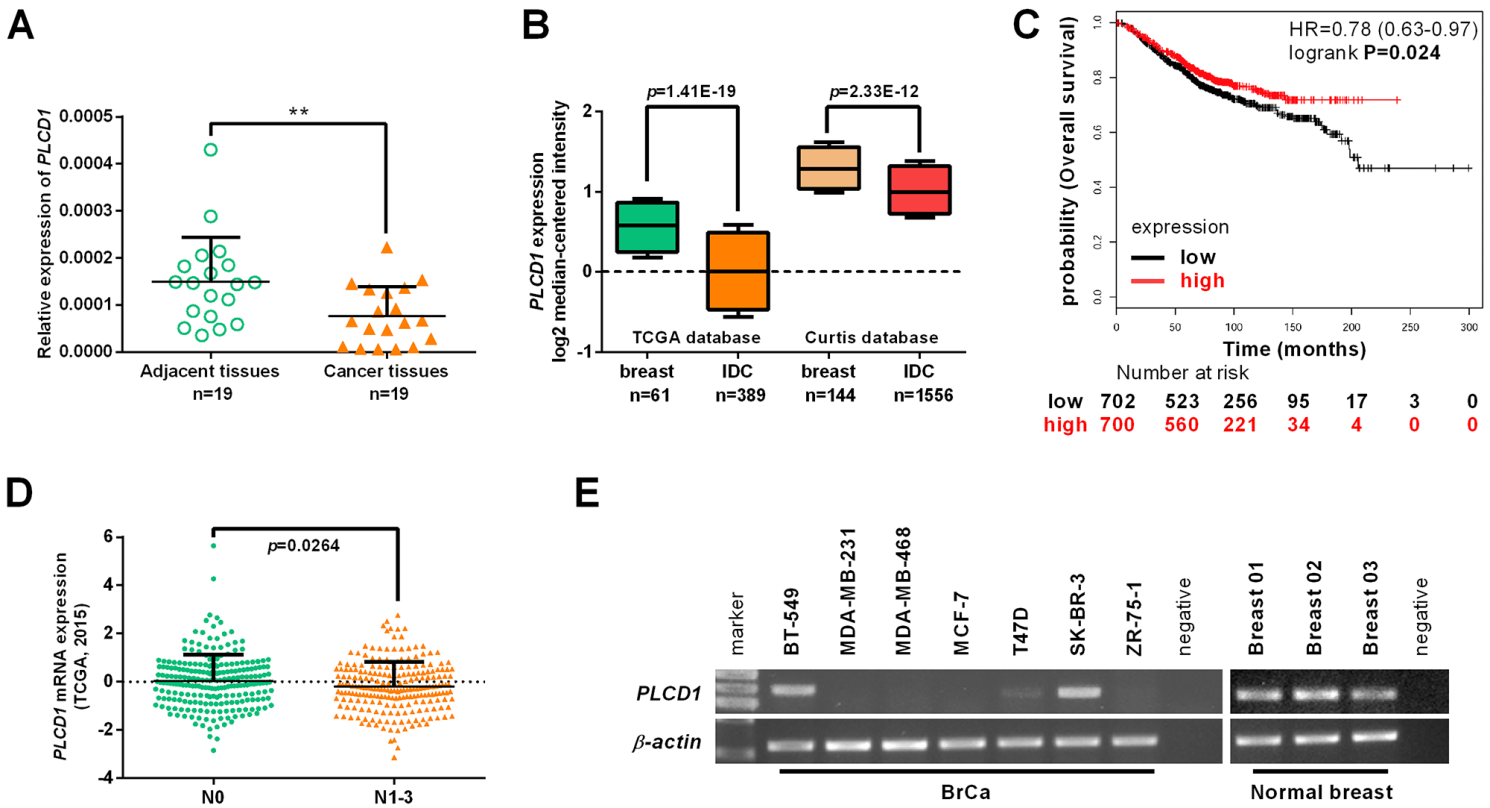
Expression of PLCD1 in breast cancer cell lines and breast cancers **(A)** Expression of *PLCD1* in a panel of breast cancer tissues matched with adjacent normal breast tissue samples measured by quantitative RT-PCR with *β-actin* as an internal control. Data were based on at least three independent assays. Means ± SD, n = 19, **p<0.01. **(B)** Expression of *PLCD1* (log2 median-centered intensity) in normal breast tissues and invasive ductal carcinomas (IDC) analyzed using the Oncomine microarray database, The Cancer Genome Atlas (TCGA) and the Curtis microarray database. **(C)** The prognostic value of *PLCD1* expression on overall survival (OS) analyzed by Kaplan-Meier Plotter (http://www.kmplot.com) in breast cancers (hazard ratio [HR] = 0.78 (0.63−0.97), *p* = 0.024). **(D)** Expression of *PLCD1* in N0 (Lymph node without metastasis, n = 232) and N1-3 (Lymph node with metastasis, n = 226) breast cancers was analyzed in The Cancer Genome Atlas (TCGA) database using the cBioPortal for Cancer Genomics (http://www.cbioportal.org/; p = 0.0264). **(E)** Expression of *PLCD1* in a panel of breast cancer cells and three normal breast tissues was detected by RT-PCR with *β-actin* as an internal control.

Expression of *PLCD1* is downregulated in breast cancer cell lines and primary breast cancers following aberrant hypermethylation of its promoter [9, 10]. In this study, expression of *PLCD1* was detected in a panel of breast cancer tissues that were matched with non-cancerous adjacent breast tissue samples, but *PLCD1* was markedly downregulated in breast cancer tissue (Figure 1A). In addition, expression of *PLCD1* was analyzed using the Oncomine microarray database (http://www.oncomine.org), and *PLCD1* was also found to be downregulated in invasive ductal carcinoma (IDC) compared with normal breast tissue (Figure 1B). Furthermore, the relationship between *PLCD1* expression and overall survival (OS) in breast cancer patients was analyzed using Kaplan-Meier Plotter (http://www.kmplot.com) for breast cancers [15]. The results showed that OS was higher when *PLCD1* is more highly expressed (hazard ratio [HR] = 0.78 (0.63−0.97), *p* = 0.024; Figure 1C). Also, expression of *PLCD1* in N0 (Lymph node without metastasis, n = 232) and N1-3 (Lymph node with metastasis, n = 226) breast cancers was analyzed using cBioPortal for Cancer Genomics (http://www.cbioportal.org/) within The Cancer Genome Atlas (TCGA) database, and the expression of *PLCD1* was much higher in N0 breast cancers compared with N1-3 breast cancers (*p* = 0.0264) (Figure 1D).

In this study, expression of *PLCD1* was also detected in a panel breast cancer cell lines and three normal breast tissues by RT-PCR. Expression was downregulated in MDA-MB-231, MDA-MB-468, MCF-7, T47D and ZR-75-1 cells, but not in BT-549 or SK-BR-3 cells, or in three normal breast tissues (Figure 1E).

### PLCD1 inhibits cell migration and invasion *in vitro*

**Figure 2:**
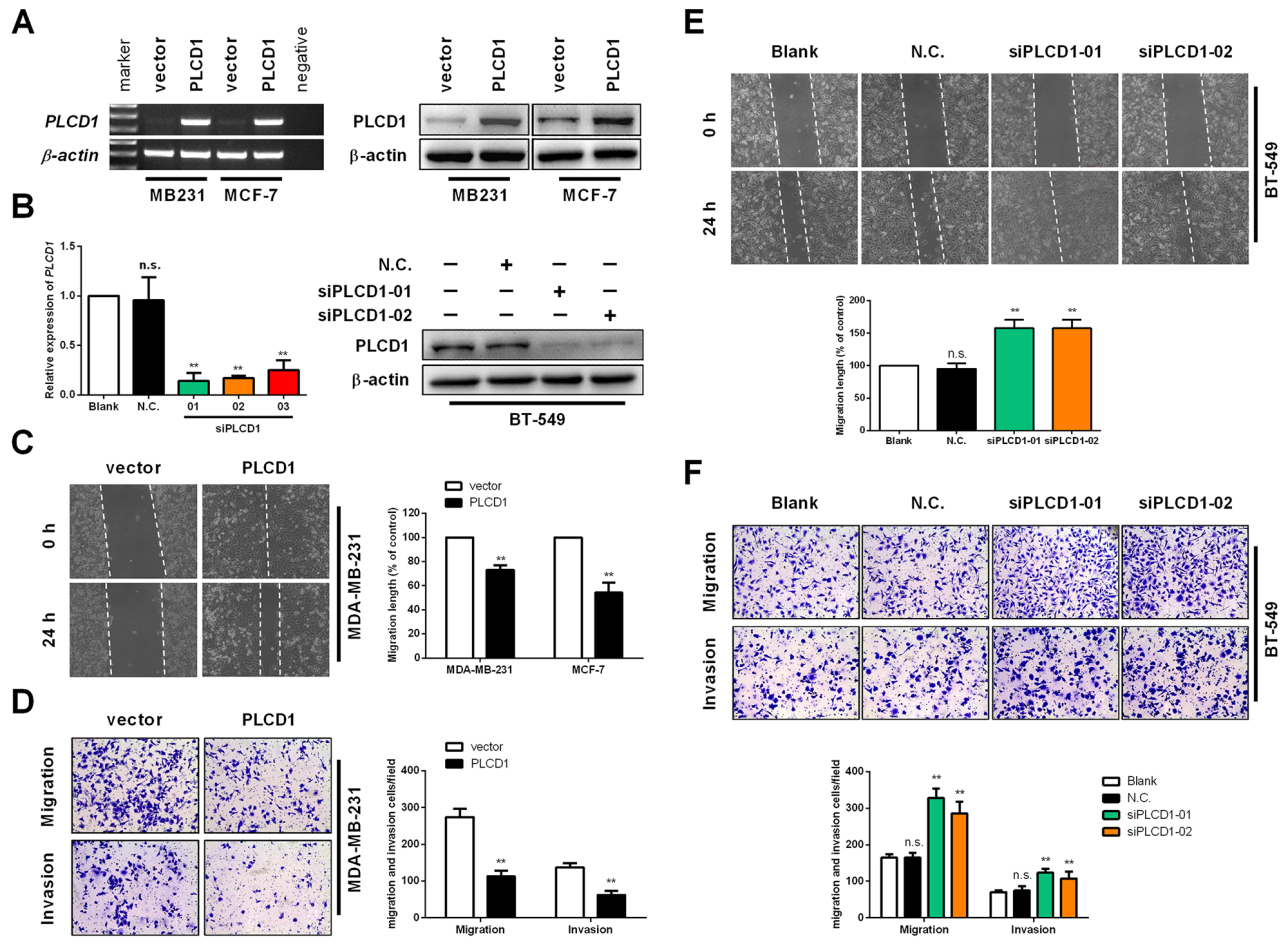
PLCD1 inhibits cell migration and invasion *in vitro* **(A)** MDA-MB-231 and MCF-7 cells transfected with vector and PLCD1 plasmid at 2 weeks after selection with geneticin. Expression of PLCD1 was measured by both RT-PCR and immunoblotting. **(B)** BT-549 cells transfected with siRNAs siN.C. and siPLCD1 at 72 h. Expression of PLCD1 was measured by both quantitative RT-PCR and immunoblotting. **(C)** Confluent monolayers of MDA-MB-231 and MCF-7 cells expressing PLCD1 (or vector-only control cells) were scratched and migration distance measured over a 24 h period by phase-contrast microscopy. Representative wound healing assays are shown on the left. Means ±SD, **p<0.01. **(D)** PLCD1/MDA-MB-231 cells and vector-only controls cells migrated to the lower side of the chamber or invaded through the Matrigel barrier to the lower side of the chamber. Cells were fixed, stained and counted by phase-contrast microscopy. Five random fields were taken for each chamber. Representative cell migration assays and cell invasion assays are shown on the left. Means ±SD, **p<0.01. **(E)** Confluent monolayers of BT-549 cells (wild type, siN.C., siPLCD1-01, and siPLCD1-02) were scratched and migration measured by phase-contrast microscopy. Means ±SD, **p<0.01. **(F)** BT-549 cells (wild type, siN.C., siPLCD1-01, siPLCD1-02) after migration or invasion. Cells in the lower side of the chamber were fixed, stained, and counted in five random fields for each chamber. Means ±SD, **p<0.01.

MDA-MB-231 and MCF-7 were used for PLCD1 expression analysis, and BT-549 cells were used for PLCD1 knockdown experiments. Expression of PLCD1 was measured in MDA-MB-231, MCF-7 and BT-549 cells at 48–72 h after transfection. PLCD1 was successfully expressed in MDA-MB-231 and MCF-7 cells (Figure 2A), and PLCD1 was clearly knocked down by siRNA in BT-549 cells, and siPLCD1-01 and 02 were chosen for subsequent assays (Figure 2B).

As demonstrated previously, cell migration is suppressed in breast cancer cells expressing PLCD1 [9, 10]. In the present study, PLCD1 was expressed in MDA-MB-231 and MCF-7 cells, and knocked down in BT-549 cells, to evaluate its function in cell migration and invasion. Wound-healing assays (WHA) and transwell assays were used to measure cell migration, and transwell assays with a Matrigel barrier were used to evaluate cell invasion. WHA results showed that cell migration was suppressed by PLCD1 in both MDA-MB-231 and MCF-7 cells (Figure 2C). By contrast, cell migration was enhanced when PLCD1 was knocked down (Figure 2E). Transwell assays without a Matrigel barrier showed the same effect in MDA-MB-231 and BT-549 cells (Figure 2D, 2F), but not in MCF-7 cells since they cannot pass through the membrane (data not shown). In addition, cell invasion was decreased in MDA-MB-231 cells expressing PLCD1 (Figure 2D), and increased when PLCD1 was knocked down in BT-549 cells (Figure 2F). Taken together, these results indicate that PLCD1 functions as a tumour suppressor affecting cell migration and invasion in breast cancer.

### PLCD1 inhibits ERK1/2/β-catenin/MMP7 signalling in breast cancer

**Figure 3:**
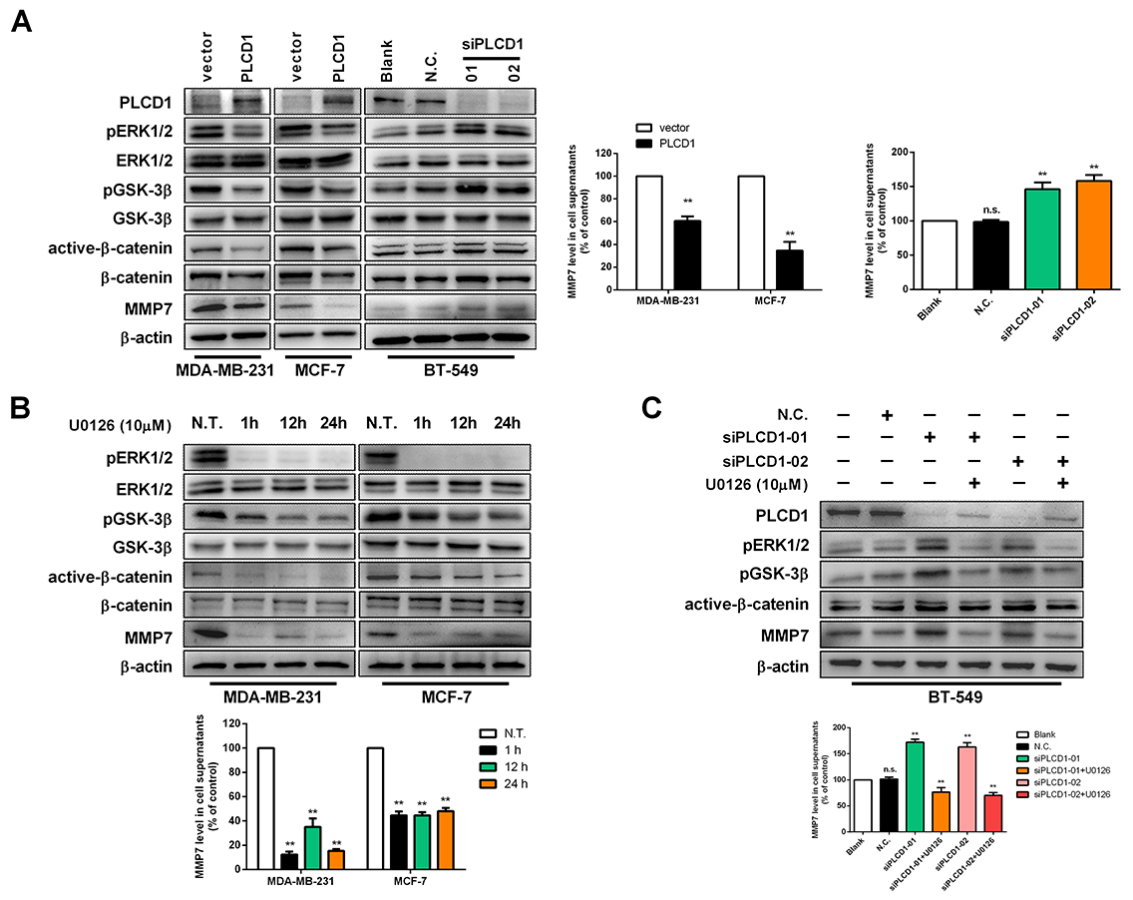
PLCD1 suppresses ERK1/2/β-catenin/MMP7 signalling **(A)** PLCD1-expressing cells and vector-only control cells (MDA-MB-231 and MCF-7) and BT-549 cells transfected with siN.C. and siPLCD1 for 72 h. PLCD1, pERK1/2, ERK1/2, pGSK-3β, GSK-3β, active-β-catenin, β-catenin, and MMP7 protein levels were measured by immunoblotting with β-actin as an internal control, and supernatant MMP7 levels were also measured by ELISA. Data were based on at least three independent assays, and representative images were shown on the left. Means ±SD, **p<0.01. **(B)** Expression of related proteins was detected by immunoblotting at the indicated time points (0 h, 1 h, 12 h, 24 h) after treatment with U0126 in MDA-MB-231 and MCF-7 cells. Supernatant MMP7 levels were also measured by ELISA. Data were based on at least three independent assays, and representative images were shown. Means ±SD, **p<0.01. **(C)** Cells transfected with siN.C. or siPLCD1 were treated with or without U0126 for 1 h, and protein levels were measured by immunoblotting in BT-549 cells. Supernatant MMP7 levels were also measured by ELISA. Data were based on at least three independent assays, and representative images were shown. Means ±SD, **p<0.01.

PLCD1 inhibits MMP7 expression, resulting in anti-tumour effects in gastric and breast cancers [5, 10], but the underlying mechanism remains elusive. In this study, immunoblotting was used to evaluate the effect of PLCD1 expression on ERK1/2, GSK-3β, β-catenin and MMP7 protein levels. ELISA was used to measure MMP7 levels in the supernatant. The results showed that PLCD1 suppressed phosphorylation of ERK1/2 and GSK-3β, and lowered levels of active-β-catenin and MMP7 in MDA-MB-231 and MCF-7 cells (Figure 3A), and the opposite effect was observed when PLCD1 was knocked down in BT-549 cells (Figure 3A). Additionally, ELISA and immunoblotting results for MMP7 in the supernatant were similar (Figure 3A).

Upon the addition of the MEK1/2 inhibitor U0126 that inhibits phosphorylation of ERK1/2, a decrease in pGSK-3β, active-β-catenin and MMP7 was observed, and this decrease was time-dependent (as shown by 1 h, 12 h and 24 h immunoblotting time points in Figure 3B). The level of MMP7 in the supernatant was decreased following U0126 treatment (Figure 3B), and U0126 also decreased pERK1/2, pGSK-3β, active-β-catenin and MMP7 when PLCD1 was knocked down in BT-549 cells. Interestingly, U0126 upregulated the expression of PLCD1 slightly (Figure 3C).

**Figure 4:**
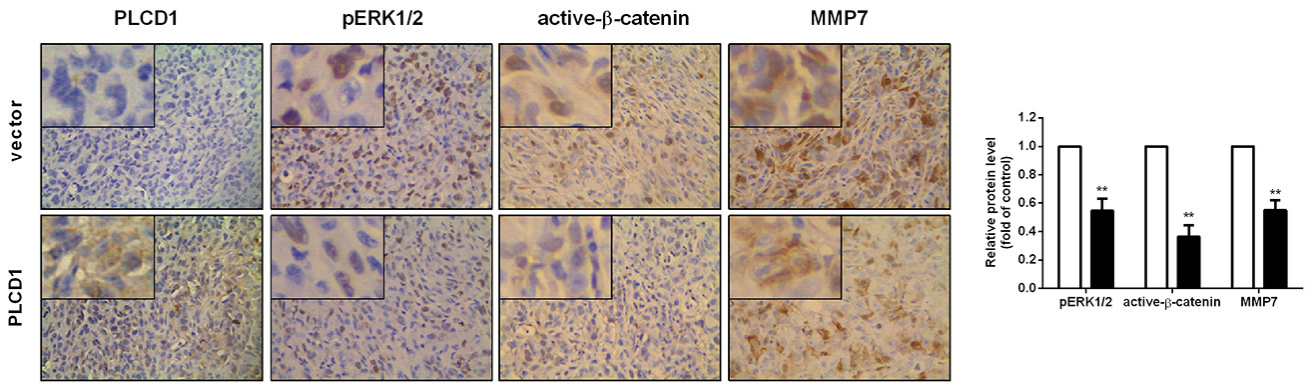
Expression of PLCD1, pERK1/2, active-β-catenin and MMP7 in MDA-MB-231 xenografts in nude mice (magnification ×400) Data were based on at least three independent assays, and representative images were shown in the left panel. Means ± SD, **p<0.01.

We constructed xenografts in nude mice using PLCD1-expressing and vector-only MDA-MB-231 cells as described previously [10]. In the present study, PLCD1, pERK1/2, active-β-catenin and MMP7 protein levels were measured by immunohistochemistry (IHC) in these tumours. The results showed that PLCD1 overexpression was accompanied by a decrease in pERK1/2, active-β-catenin and MMP7 (Figure 4).

### PLCD1 suppresses KIF3A in breast cancer

**Figure 5:**
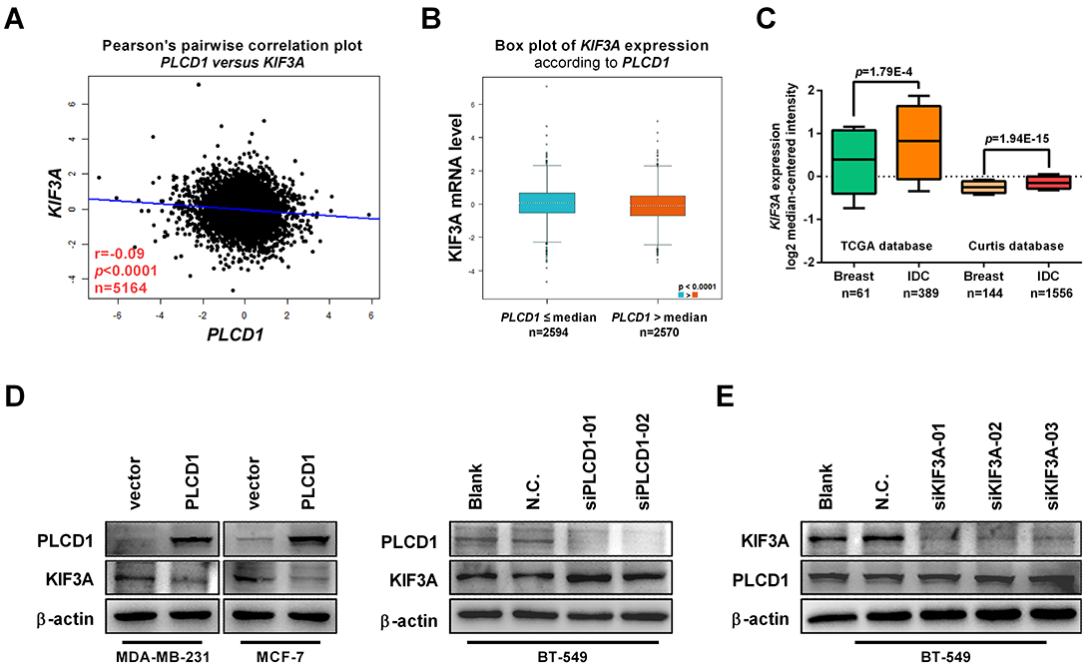
PLCD1 suppresses KIF3A in breast cancer **(A)** Correlation between the expression of *PLCD1* and *KIF3A* in breast cancer analyzed by Breast Cancer Gene-Expression Miner v4.0 (bc-GenExMiner v4.0) (r = −0.09, *p*<0.0001, n = 5164). **(B)** Expression of *KIF3A* in cells expressing high (*PLCD1*>median) and low (*PLCD1*≤median) amounts of *PLCD1* (*p*<0.0001). **(C)** Expression of *KIF3A* (log2 median-centered intensity) in normal breast tissues and invasive ductal carcinomas (IDC) analyzed using the Oncomine microarray database, the Cancer Genome Atlas (TCGA) and the Curtis microarray database. **(D)** PLCD1-expressing cells and vector-only control cells (MDA-MB-231, MCF-7) and BT-549 cells transfected with siN.C. and siPLCD1 for 72 h. KIF3A protein levels were measured by immunoblotting with β-actin as an internal control. Data were based on at least three independent assays, and representative images were shown. **(E)** BT-549 cells transfected with siN.C. and siKIF3A for 72 h. KIF3A and PLCD1 protein levels were measured by immunoblotting with β-actin as an internal control. Data were based on at least three independent assays, and representative images were shown.

From the above results, we concluded that PLCD1 inhibits cell migration and invasion by suppressing, at least in part, ERK1/2/β-catenin/MMP7 signalling. However, the mechanism of this suppression is not understood, and we speculated that there may be one or more factors mediating this negative regulation. Based on an analysis of potential PLCD1-interacting proteins in the BioGRID3.4 database (https://thebiogrid.org), kinesin family member 3A (KIF3A) identified and linked to PLCD1 for the first time in this context (Supplementary Figure 1). The correlation between the expression of *PLCD1* and *KIF3A* was analyzed using bc-GenExMiner v4.0 (http://bcgenex.centregauducheau.fr) (r = −0.09, *p*<0.0001, n = 5164; Figure 5A) [16, 17]. Moreover, the expression of *KIF3A* was lower in the group with relatively high expression of *PLCD1* (Figure 5B). The expression of *KIF3A* was analyzed using the Oncomine microarray database, and *KIF3A* was found to be increased in breast cancers compared with normal breast tissues (Figure 5C). Next, we investigated the effect of PLCD1 expression on KIF3A regulation by immunoblotting and found that KIF3A was inhibited by PLCD1 in MDA-MB-231 and MCF-7 cells, but stimulated when PLCD1 was knocked down in BT-549 cells (Figure 5D). KIF3A was also knocked down by siRNA without any effect on the expression of PLCD1 in BT-549 cells (Figure 5E). These results suggest that KIF3A expression was suppressed by PLCD1, and it may therefore act as a downstream mediator of PLCD1.

### Knockdown of KIF3A suppresses cell migration and invasion

**Figure 6:**
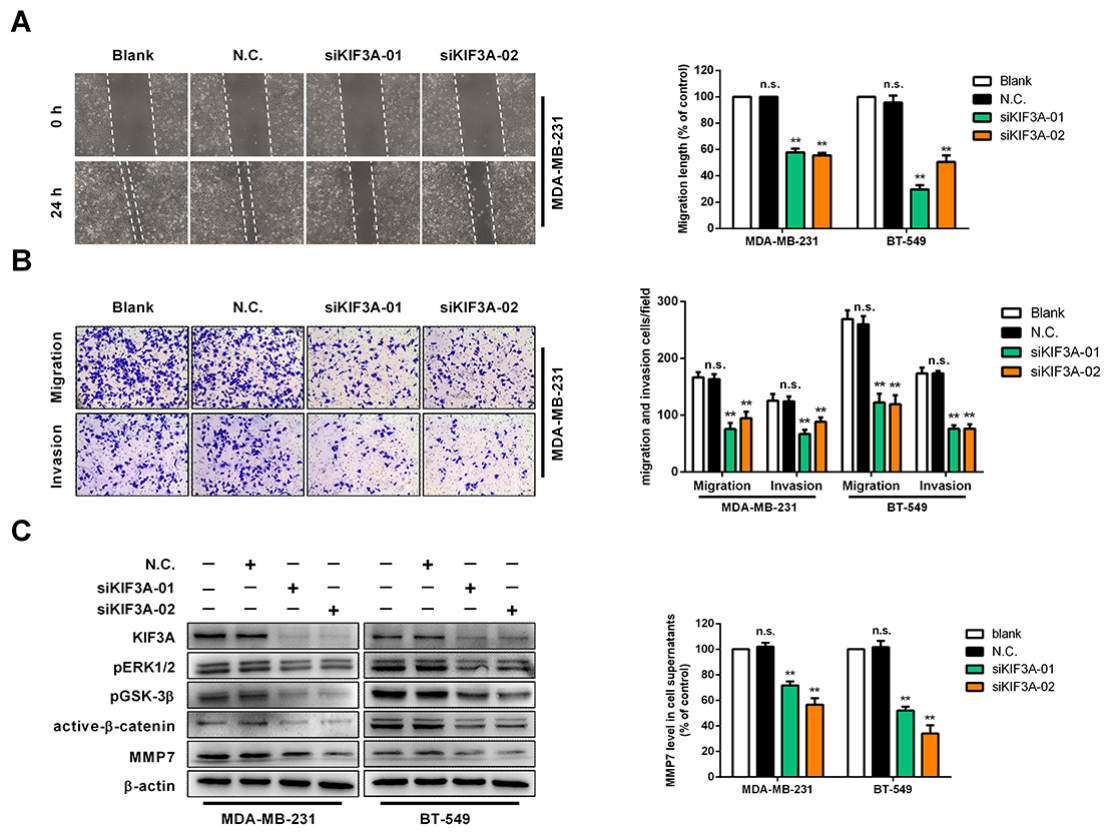
Knockdown of KIF3A suppresses cell migration and invasion *in vitro* **(A)** Confluent monolayers of MDA-MB-231 or BT-549 cells were transfected with siN.C. or siKIF3A, scratched, and migration distance measured over a 24 h period using an inverted microscope. Representative wound-healing assays are shown on the left. Means ±SD, **p<0.01. **(B)** MDA-MB-231 cells and BT-549 cells transfected with siN.C. and siPLCD1 migrated to the lower side of the chamber or invaded through the Matrigel barrier. Cells were fixed, stained and counted using an inverted microscope. Five random fields were taken for each chamber. Representative cell migration and cell invasion assays are shown on the left. Means ±SD, **p<0.01. **(C)** MDA-MB-231 and BT-549 cells were transfected with siN.C. and siKIF3A for 72 h and KIF3A, pERK1/2, pGSK-3β, active-β-catenin, and MMP7 protein levels were measured by immunoblotting with β-actin as an internal control. Supernatant MMP7 levels were also measured by ELISA. Data were based on at least three independent assays, and representative images were shown on the left. Means ±SD, **p<0.01.

Next, we explored whether KIF3A had an effect on regulating cell migration, cell invasion and ERK1/2/β-catenin/MMP7 signalling. Expression of KIF3A was knocked down to evaluate the function of KIF3A on cell migration and invasion in MDA-MB-231 and BT-549 cells. WHA and transwell assays showed that cell migration was decreased in MDA-MB-231 and BT-549 cells (Figure 6A, 6B). Cell invasion was suppressed in transwell chambers with a Matrigel barrier (Figure 6B), and pERK1/2, pGSK-3β, active-β-catenin and MMP7 protein levels were decreased after knocking down KIF3A in MDA-MB-231 and BT-549 cells, as were MMP7 levels in the supernatant (Figure 6C). These results indicated that KIF3A promotes cell migration and invasion by regulating, at least in part, pERK1/2/active-β-catenin/MMP7 signalling.

### KIF3A mediates PLCD1 tumour suppression activity in breast cancer

**Figure 7:**
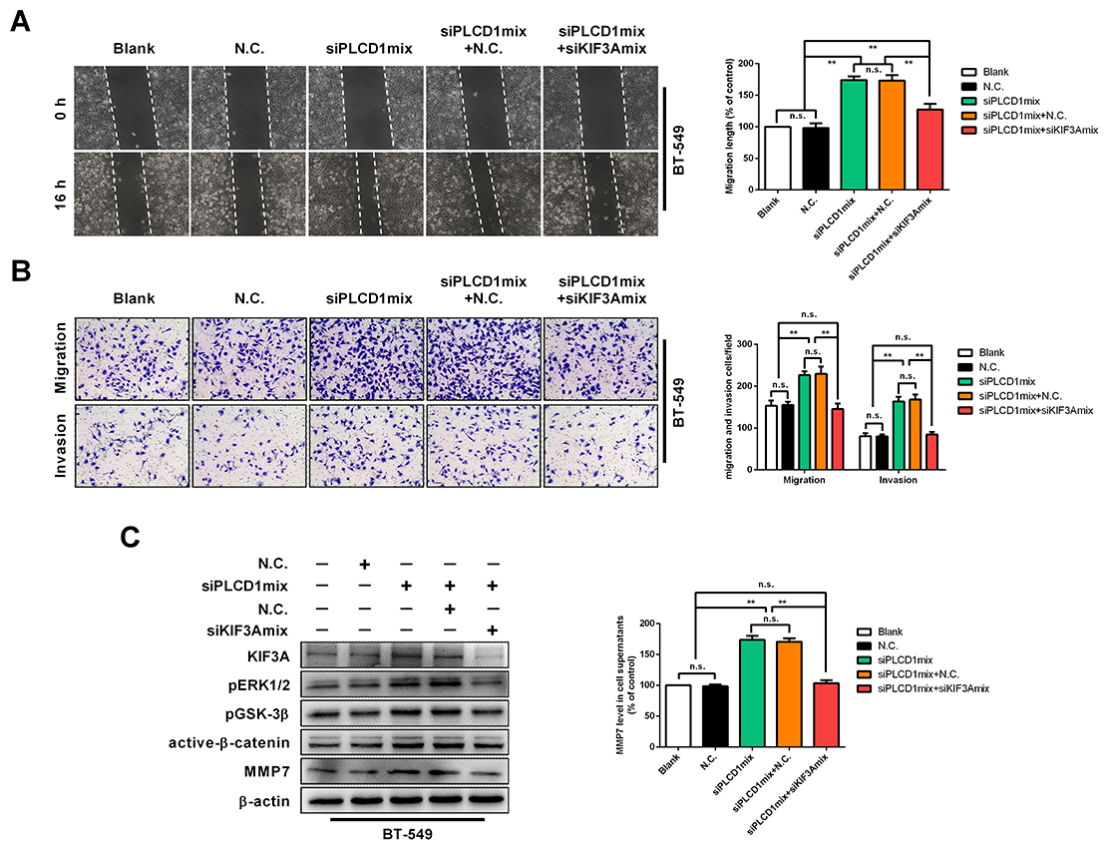
KIF3A acts as a downstream mediator of PLCD1 BT-549 cells transfected with siN.C. or siPLCD1 were also transfected simultaneously with (or without) siKIF3A for 72 h. **(A)** Cell migration assays. Confluent cell monolayers were scratched and migration distance was measured over a 16 h period by phase-contrast microscopy. Representative wound-healing assays are shown on the left. Means ± SD, **p<0.01. **(B)** Cell invasion assays. BT-549 cells migrated to the lower side of the chamber or invaded through the Matrigel barrier. Cells were fixed, stained and counted using an inverted microscope. Five random fields were taken for each chamber. Representative cell migration and cell invasion assays are shown on the left. Means ±SD, **p<0.01. **(C)** Immunoblotting of KIF3A, pERK1/2, pGSK-3β, active-β-catenin, and MMP7 protein levels with β-actin as an internal control. Supernatant MMP7 levels were also measured by ELISA. Data were based on at least three independent assays, and representative images were shown. Means ± SD, **p<0.01.

To address whether KIF3A mediated PLCD1-based regulation of pERK1/2/active-β-catenin/MMP7 signalling, PLCD1 and KIF3A were knocked down in BT-549 cells simultaneously. WHA and transwell assays showed that cell migration was increased by knocking down PLCD1, and decreased further by simultaneously knocking down KIF3A (Figure 7A, 7B). This was also the case for cell invasion in transwell assays (Figure 7B). In addition, knocking down KIF3A suppressed pERK1/2, pGSK-3β, active-β-catenin and MMP7 protein levels induced by knocking down PLCD1 in BT-549 cells (Figure 7C).

## DISCUSSION

Chromosome 3p, including the 3p22 region, is frequently deleted in multiple cancers [18]. This region includes the candidate tumour suppressor *PLCD1*, the promoter of which is frequently aberrantly hypermethylated in breast cancers [9, 10]. PLCD1 has been demonstrated to suppress MMP7 levels and thereby regulate cell migration in high-stage gastric and breast cancers [5, 10]. However, the underlying mechanism remains largely unknown. In this study, we clarify the mechanism and found that KIF3A and ERK1/2/β-catenin/MMP7 signalling are involved in the inhibition of cell migration and invasion by PLCD1.

PLCD1 is an enzyme involved in various aspects of cell migration, cell invasion and metastasis, cell proliferation, cytoskeletal organization, receptor endocytosis, and hormone secretion, mainly through direct interaction with the small GTPase Ral and calmodulin [19, 20]. In oesophageal squamous cell carcinoma (ESCC), PLCD1 was first reported as a tumour suppressor following single nucleotide polymorphism-mass array experiments in which PLCD1 suppressed cell cycle progression and cell migration [6]. In high-stage gastric cancers, PLCD1 regulates cell proliferation, and migration and metastasis *in vivo* [5]. Moreover, in breast cancer, we demonstrated that PLCD1 inhibits cell proliferation and cell migration [10]. Furthermore, PLCD1 inhibits the epithelial-mesenchymal transition (EMT) of KRAS-mutated colorectal cancers [7]. Mounting evidence therefore suggests that PLCD1 functions as a tumour suppressor. Consistent with this viewpoint, in the present study, PLCD1 was downregulated in breast cancers. Relatively low expression levels are correlated with a poor prognosis in breast cancers. Also, PLCD1 suppresses cell migration and invasion in breast cancers.

It is interesting that MMP7 protein levels were inhibited by PLCD1 in gastric and breast cancer in our previous work [5], even though the underlying mechanism is not known. MMP7 is a matrix metallopeptidase that is involved in tumour progression by influencing cell invasion, metastasis, and the epithelial-to-mesenchymal transition [21–23]. It is well known that MMP7 is a downstream target of the Wnt/β-catenin signalling pathway that is activated in multiple cancers including breast cancers [24]. In this study, PLCD1 negatively regulated pGSK-3β, active-β-catenin and MMP7, suggesting for the first time that the β-catenin signalling pathway may be involved in the anti-tumour activity of PLCD1.

It has been demonstrated that there exists crosstalk between the Wnt/β-catenin signalling pathway and several other signalling pathways in breast cancer, including those involving MAPKs and Akt, suggesting inhibition of β-catenin may depend on MAPKs and/or Akt signalling [25, 26]. Indeed, PLCD1 induces cell cycle arrest by inhibiting Akt signalling in ESCC [6], and suppresses EMT through ERK signalling in KRAS-mutated colorectal cancers [7]. In the present study, PLCD1 suppressed phosphorylation of ERK but did not affect Akt phosphorylation in breast cancer (data not shown). Thus, the suppressive effect of PLCD1 may depend on crosstalk between β-catenin signalling and ERK signalling in breast cancer. Moreover, inhibition of the phosphorylation of ERK using the inhibitor U0126 indicated that β-catenin signalling acts downstream of ERK in this context. Interestingly, inhibition of ERK phosphorylation increased the expression of PLCD1, consistent with a previous report describing a regulatory loop between PLCD1 and phosphorylated ERK [7].

It is not clear how PLCD1 regulates ERK phos-phorylation. It has been reported that E-cadherin engagement leads to ERK inhibition in a PI3K/Akt-dependent pathway in differentiating intestinal epithelial cells [27], and in KRAS-mutated colorectal cancers, suggesting PLCD1 may suppress phosphorylation of ERK by engaging E-cadherin [7]. However, the fact that PLCD1 had no effect on Akt phosphorylation in breast cancer remains puzzling. Analysis of potential PLCD1-interacting proteins using BioGRID3.4 (https://thebiogrid.org) identified KIF3A, which is required for ciliogenesis and has been linked to various cancers [28, 29]. KIF3A promotes cell proliferation and invasion through the KIF3A-DVL2-β-catenin axis in advanced prostate cancer [30]. However, in non-small cell lung cancer (NSCLC), KIF3A binds to β-arrestin and suppresses Wnt/β-catenin signalling [31]. Thus, the significance and mechanism of KIF3A in breast cancer is still unclear. In this study, there was a negative correlation between the expression of PLCD1 and KIF3A in breast cancer. Moreover, knockdown of KIF3A suppressed pERK, pGSK-3β, active-β-catenin and MMP7 protein levels, which mitigated the upregulation of these proteins when PLCD1 was simultaneously knocked down. Therefore, KIF3A was demonstrated to mediate the effect of PLCD1 on ERK1/2/β-catenin/MMP7 signalling, at least in part, in breast cancer.

In conclusion, the present study provides evidence that ERK1/2/β-catenin/MMP7 signalling is involved in PLCD1-based regulation of cell migration and invasion, and KIF3A acts as a mediator in the attenuation of ERK1/2/β-catenin/MMP7 signalling. These results expand our knowledge of PLCD1 and downstream signalling in breast cancer.

## MATERIALS AND METHODS

### Cell lines and primary breast samples

Seven breast cancer cell lines (BT-549, MDA-MB-231, MDA-MB-468, MCF-7, T47D, SK-BR-3, and ZR-75-1) were studied [32]. MDA-MB-231 cells were maintained in DMEM (Gibco-BRL, Karlsruhe, Germany), and the others were maintained in RPMI 1640 medium (Gibco-BRL) supplemented with 10% fetal bovine serum (Gibco-BRL), and maintained at 37°C with 5% CO_2_. Primary breast tumour samples, matched non-cancer tissues, and normal breast tissues were obtained from the First Affiliated Hospital of Chongqing Medical University between January 2015 and October 2015. All samples underwent a histopathological diagnosis by pathologists. All patients provided written consent, and the study was approved by the Ethics Committee of the First Affiliated Hospital of Chongqing Medical University.

### Plasmids, siRNAs and transfection

PLCD1-expressing and control plasmids have been reported previously [9, 10]. PLCD1 siRNA and KIF3A siRNA kits were purchased from RIBOBIO (Guangzhou, China). All transfections were performed using Lipofectamine 2000 (Invitrogen, Carlsbad, CA, USA) according to the manufacturer's instructions with a concentration of 4μg plasmid or 50 nM siRNA. Transfected cells were harvested for subsequent assays at 48 h/72 h after transfection.

### RNA extraction

RNA extraction was performed as previously described [33]. Briefly, total RNA was extracted using TRIzol reagent (Invitrogen), the RNA concentration was measured by NanoDrop 2000 spectrophotometer (Thermo Scientific), and RNA quality was determined by gel electrophoresis.

### Reverse transcription, semi-quantitative PCR and quantitative PCR

Reverse transcription and semi-quantitative RT-PCR were performed using Go-Taq polymerase (Promega, Madison, WI, USA), and a SYBR Green PCR Master Mix kit (Invitrogen) for quantitative RT-PCR, with an Applied Biosystems 7500 (Applied Biosystems, Foster City, CA, USA) and *β-actin* as an internal control [34]. Relative expression was determined using the 2^(−△Ct)^ method. Primers used are listed in Supplementary Table 1.

### Wound-healing and transwell assays

Cell migration was measured using wound-healing and transwell assays, and cell invasion was also evaluated by transwell assay with Matrigel (BD Biosciences, San Jose, CA, USA) as a barrier [35]. Briefly, 2×10^6^ cells/well were plated in six-well plates 1 day before scratching using a sterile tip. Migration distance was measured under phase-contrast microscopy (Leica DMI4000B, Milton Keynes, Bucks, UK) at the indicated time point. Moreover, 2×10^4^ cells were seeded into transwell chambers (Corning Life Science, Bedford, MA, USA) with or without Matrigel, and after 16–24 hours, cells on the lower side of the chamber were counted after fixing with 4% paraformaldehyde and staining with 0.5% crystal violet under phase-contrast microscopy (Leica).

### Immunoblotting

Cells were washed twice with cold 1×PBS, and total lysates were extracted with RIPA lysis buffer (Beyotime Institute of Biotechnology, Shanghai, China) containing protease inhibitor cocktail (Pierce, Cramlington, UK) with mixing by sonication. The lysate concentration was measured using a bicinchoninic acid (BCA) standard curve. Lysates (40μg) were heated at 95°C for 5 min and separated by 10–12% sodium dodecyl sulphate polyacrylamide gel electrophoresis (SDS-PAGE). After transferring onto polyvinylidene fluoride (PVDF) membranes and blocking with 5% milk, membranes were incubated with primary antibodies against PLCD1 (ab134936), MMP7 (ab207299; Abcam, Cambridge, MA, USA), KIF3A (#8507S), ERK1/2 (#4695), pERK1/2 (#4370), β-catenin (#8480), GSK-3β (#9315), pGSK-3β (#9323), β-actin (#4970; Cell Signaling Technology, Danvers, MA, USA), or active-β-catenin (05-665; Merck Millipore, Billerica, MA, USA) overnight, followed by secondary antibodies (1:5000 dilution, Cell Signaling Technology). Membranes were visualized using ECL Plus Detection Reagents (RPN2132, GE Healthcare Life Science, Amersham, UK).

### Immunohistochemistry

Standard streptavidin-peroxidase immunohisto-chemistry procedures were performed following the manufacturer's instructions with an UltraSensitive TM SP Kit (Maixin-Bio, Fujian, China) as previously described [36]. Primary antibodies against PLCD1 (ab134936; Abcam), MMP7 (ab207299; Abcam), pERK1/2 (#4370; Cell Signaling Technology), or active-β-catenin (05-665; Millipore) were employed, and PBS was used as a negative control. All immunohistochemical images were subjected to mean optical density (OD) measurement using Image Pro Plus (IPP, version 6.0; Media Cybernetics, Silver Spring, MD, USA).

### ELISA

MMP7 levels in supernatants were measured using ELISA kits (R&D Systems, Inc, Minneapolis, MN) following the manufacturer's instructions [37]. Briefly, standards and supernatants were added to wells coated with antibody against MMP7 and incubated for 2 h at room temperature. The intensity was measured at 450 nm.

### Statistical analysis

All data were acquired at least in triplicate and are presented as the mean ± standard deviation (SD). Values were compared with controls using the two-tailed Student *t*-test in Prism 6 software (GraphPad Software, Inc., La Jolla, CA, USA), and *p*<0.05 was considered significant.

## SUPPLEMENTARY MATERIALS FIGURES AND TABLES


