# Supplementary material for: Phospholipase Cδ1 suppresses cell migration and invasion of breast cancer cells by modulating KIF3A-mediated ERK1/2/β- catenin/MMP7 signalling
Source: Oncotarget. 2017 Mar 10;8(17):29056–66. doi: 10.18632/oncotarget.16072 (PMC5438712; doi:10.18632/oncotarget.16072)
Supplement: Supplementary file 1 [file oncotarget-08-29056-s001.pdf]

Phospholipase Cδ1 suppresses cell migration and invasion of breast cancer cells by modulating KIF3A-mediated ERK1/2/β-catenin/MMP7 signalling

SUPPLEMENTARY MATERIALS

SUPPLEMENTARY FIGURE AND TABLE

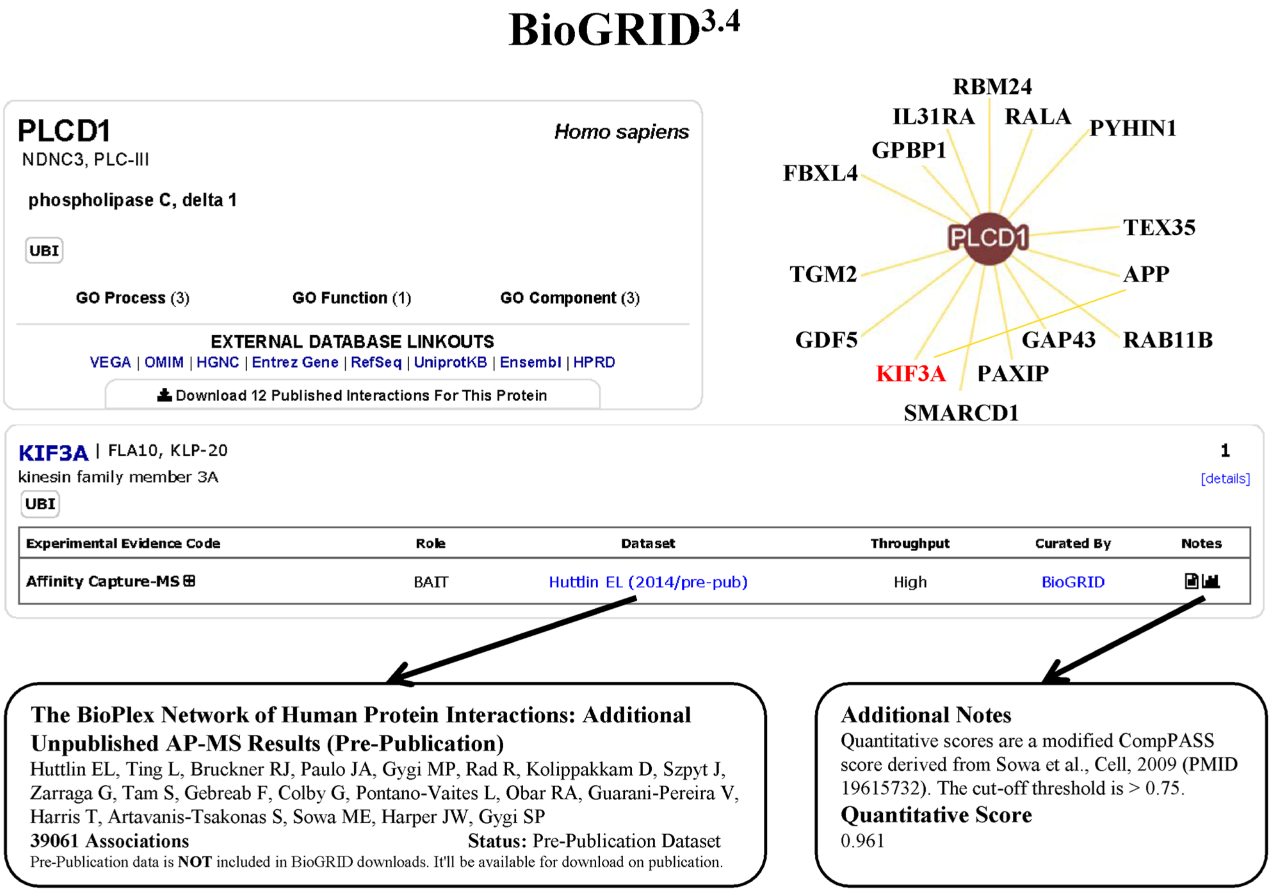

Supplementary Figure 1: PLCD1 interacts with KIF3A. Potential PLCD1-interacting partners were analyzed using BioGRID3.4 (https://thebiogrid.org). A total of 15 potential interactors were identified, and kinesin family member 3A (KIF3A) was identified in the Huttlin EL dataset (The BioPlex Network of Human Protein Interactions: Additional unpublished AP-MS results, pre-publication) with a quantitative score of 0.961.

Supplementary Table 1: PCR primers sequences and reaction conditions

PCR	Primers	Sequence (5`-3`)	Size (bp)	T_A(°C)	cycle
RT-PCR					
	PLCD1 F	TGTCGCTACTCAAGTGAGTC	197	55	32
	PLCD1 R	AGTCCTCCTGCAACTTGTAG			
	β-actin F	TCCTGTGGCATCCACGAAACT	315	55	23
	β-actin R	GAAGCATTTGCGGTGGACGAT			
Real-time PCR					
	PLCD1 F	TGTCGCTACTCAAGTGAGTC	197	60	40
	PLCD1 R	AGTCCTCCTGCAACTTGTAG			
	KIF3A F	CTGATATCAGTGGGTCAGAGGA	262	60	40
	KIF3A R	TCCAGCAAAGACTGATGCTCT			
	β-actin F	TCCTGTGGCATCCACGAAACT	315	60	40
	β-actin R	GAAGCATTTGCGGTGGACGAT			

T_A, Annealing Temperature
